# The serine-arginine-rich protein PfSR-X2 modulates human malaria parasite gene expression during the intraerythrocytic developmental cycle

**DOI:** 10.3389/fcimb.2026.1842355

**Published:** 2026-05-26

**Authors:** Ye Hu, Chunyu Zhuang, Mengyu Li, Jiawei Wu, Lili Zhou, Minghao Li, Xinyue Li, Xuli Du, Xiaomin Shang, Yanli Bai, Dezhi Zhao, Shenbo Chen, Yanting Fan

**Affiliations:** 1 Department of Parasitology, School of Medicine, and Department of Blood Transfusion, Xi’an International Medical Center Hospital, Northwest University, Xi’an, Shaanxi, China; 2 Department of Parasitology, School of Basic Medical Science, Central South University, Changsha, Hunan, China; 3 National Key Laboratory of Intelligent Tracking and Forecasting for Infectious Diseases, National Institute of Parasitic Diseases, Chinese Center for Disease Control and Prevention (Chinese Center for Tropical Diseases Research), National Health Commission of the People’s Republic of China (NHC) Key Laboratory of Parasite and Vector Biology, World Health Organization (WHO) Collaborating Center for Tropical Diseases, National Center for International Research on Tropical Diseases, Shanghai, China

**Keywords:** ApiAP2 gene family, PfSR-X2, *Plasmodium faciparum*, post transcriptional regulation, *rif* multigene family, RNA bind protein

## Abstract

**Background:**

Malaria, caused by *Plasmodium* parasites, remains a major parasitic disease worldwide. Post-transcriptional gene regulation is essential for the intraerythrocytic development of *Plasmodium falciparum*, yet the functions of many parasite RNA-binding proteins remain poorly understood. Serine/arginine-rich proteins are important regulators of RNA metabolism in eukaryotes. In this study, we investigated the function of the SR-related protein PfSR-X2, encoded by PF3D7_0319500, during the asexual blood-stage development of *P. falciparum*.

**Methods:**

PfSR-X2 was characterized by sequence analysis, structural prediction, expression profiling, and subcellular localization assays. A glucosamine-inducible *glmS*-based conditional knockdown parasite line was generated using CRISPR-Cas9-mediated genome editing. The effects of PfSR-X2 depletion on parasite growth, developmental progression, gene expression, and alternative splicing were examined by growth assays, Giemsa-stained blood smears, and RNA sequencing. RNA immunoprecipitation followed by sequencing was further performed to identify PfSR-X2-associated transcripts.

**Results:**

PfSR-X2 was expressed throughout the intraerythrocytic developmental cycle and localized to both nuclear and cytoplasmic compartments. Attempts to disrupt *pfsr-x2* were unsuccessful, suggesting that PfSR-X2 is required for asexual blood-stage growth. Conditional depletion of PfSR-X2 resulted in reduced parasite proliferation and impaired merozoite production. Transcriptomic analysis revealed marked stage-specific changes in gene expression after PfSR-X2 knockdown, particularly affecting genes involved in RNA metabolism, antigenic variation, host-parasite interactions, and ApiAP2-associated regulatory pathways. The *rif* multigene family was strongly perturbed, whereas the overall *var* transcriptional pattern was largely unchanged. In contrast, alternative splicing changes were limited and were mainly detected at the schizont stage. RNA immunoprecipitation sequencing identified a defined subset of PfSR-X2-associated transcripts, including transcripts encoding antigenically variant proteins and ApiAP2 transcription factors.

**Conclusion:**

These findings identify PfSR-X2 as an essential RNA-associated regulator in *P. falciparum*. Rather than acting as a global splicing factor, PfSR-X2 appears to contribute mainly to stage-specific post-transcriptional regulation of transcripts associated with parasite development, antigenic variation, and transcriptional regulatory networks.

## Background

Malaria continues to impose a major global health burden, causing approximately 282 million cases and 610,000 deaths in 2024 (World Health Organization, 2025). Disease pathogenesis is linked to the obligate intracellular lifestyle of blood-stage *Plasmodium* parasites, which invade and proliferate within human erythrocytes. Successful colonization of this niche requires finely tuned regulatory networks that control parasite gene expression (Hadjimichael and Deitsch, 2025). In addition to transcriptional cascades of intraerythrocytic gene expression, post-transcriptional mechanisms help to ensure the precise, timely production of proteins essential for parasite survival and virulence (Liu et al., 2022; Tang et al., 2025; Guan et al., 2024).

Among the diverse layers of post-transcriptional control, alternative splicing is a key mechanism for proteomic expansion (Marasco and Kornblihtt, 2023). By generating multiple mRNA isoforms from a single gene, alternative splicing enhances functional diversity, thereby enabling eukaryotes to support complex development and rapidly adapt to environmental changes (Alhabsi et al., 2025). While this process is well-characterized in model eukaryotes (Du et al., 2024), its biological role in the parasitic protozoan *Plasmodium falciparum* remains enigmatic. The parasite possesses a genome of approximately 5400 genes, which, although compact, is sufficient to drive a complex multi-stage life cycle in both human and mosquito hosts. The apparent paucity of *P. falciparum* genes raises a central question: does alternative splicing confer the molecular flexibility necessary for *P. falciparum* to expand its coding capacity and orchestrate stage-specific development? Resolving this question is crucial to understanding the regulatory architecture underlying parasite adaptation and pathogenesis.

Serine/arginine-rich (SR) proteins are a principal family of RNA-binding proteins involved in alternative splicing (Li et al., 2023). Typically, the N-terminal region of an SR protein contains one or two RNA recognition motifs (RRMs), while the C-terminal domain is enriched in serine and arginine residues (Shepard and Hertel, 2009). During RNA splicing, SR proteins recognize and bind to exonic splicing enhancers (ESEs), thereby promoting the assembly of U1 and U2 small nuclear ribonucleoproteins (snRNPs) and facilitating the splicing process (Ule and Blencowe, 2019; Wilkinson et al., 2020; Wan et al., 2020). The number of SR proteins varies across species, with 12 members identified in humans and 18 in *Arabidopsis thaliana* (Zhao et al., 2021). In animals, SR proteins such as SRSF11, SRSF2, and SRSF10 are highly conserved (Richardson et al., 2011). SRSF11 has been linked to the regulation of human telomerase activity (Listerman et al., 2013), while SRSF10 plays a critical role in modulating alternative splicing events by binding to GAAA-rich motifs. Loss of SRSF10 results in defects in adipogenesis and severe underdevelopment of subcutaneous white adipose tissue, likely due to improper splicing of the seventh exon of the *Lpin1* gene (Li et al., 2014). In contrast to animals, higher plants generally possess a larger number of SR genes with unique subfamilies, such as RS, SCL, and RS2Z (Guo et al., 2025; Morton et al., 2019; Barta et al., 2010). The RS2Z subfamily, which contains two zinc finger domains, is primarily confined to land plants and highlights a distinctive evolutionary divergence in SR protein family structure and function.

The *P. falciparum* genome encodes 120 proteins that contain RNA recognition motifs (RRMs), including several regulatory SR proteins (Goyal et al., 2022; Hollin et al., 2024; Reddy et al., 2015). Functional studies on the SR protein family in *Plasmodium* have so far focused on only six members: PfSR1, PfSF1, PfSRSF4, PfSRSF12, PfSR-MG, and PfGBP2. Overexpression of PfSR1 has been shown to alter alternative splicing activity and reduce the growth rate of asexual blood-stage parasites (Goyal et al., 2021). PfGBP2, a multifunctional protein that contains an RGG motif, is involved in repressing translation without affecting mRNA levels (Edwards-Smallbone et al., 2022). Disruption of the alternative splicing factor PbSR-MG impairs sex-specific splicing events and results in a reduced ability of the parasite to differentiate into male gametocytes and oocysts, thereby lowering its transmission potential between vertebrate and insect hosts (Yeoh et al., 2019). These studies underscore the critical role of SR proteins in regulating alternative splicing in *Plasmodium*. However, much remains to be understood regarding the full spectrum of SR protein functions and their contributions to parasite development and transmission.

In this study, we investigated the function of PfSR-X2 (PF3D7_0319500), a protein homologous to the human RNA-binding motif protein, X-linked 2 (hRBMX2, AAI05583.1). Our results demonstrate that PfSR-X2 is essential for parasite growth and development throughout the intraerythrocytic developmental cycle (IDC). The knockdown of PfSR-X2 led to widespread alterations in gene expression, particularly affecting the antigenically variant *rif* multigene family and multiple ApiAP2 transcription factor genes. Although changes in alternative splicing were relatively limited, they were most pronounced at the schizont stage. Furthermore, RNA immunoprecipitation followed by sequencing (RIP-seq) revealed that PfSR-X2 directly interacts with transcripts derived from some antigenic variation gene families as well as members of the ApiAP2 transcription factor family. These findings suggest that PfSR-X2 plays a conserved role in post-transcriptional RNA regulation. Collectively, our study highlights the function of PfSR-X2 in parasite development and provides new insights that may contribute to advancing malaria research.

## Materials and methods

### Parasite culture

*Plasmodium falciparum* 3D7 and transfection lines were cultivated *in vitro* according to standard procedures. In brief, parasites were maintained in human O^+^ erythrocytes at 37 °C under a 5% CO_2_/5% O_2_ atmosphere. Cultures were sustained in RPMI 1640 medium (Thermo Fisher Scientific, Carlsbad, CA, USA) supplemented with 5 g L^−1^ Albumax I (Thermo Fisher Scientific). To obtain tightly synchronized parasites, schizonts were purified with Percoll/sorbitol reagent. Roughly 5 h after schizonts invaded fresh erythrocytes, the resulting ring-stage parasites were synchronized with 5% sorbitol treatment and used for subsequent studies.

### Plasmid construction and parasite transfection

Plasmid construction was performed as previously described (Tang et al., 2024). To generate the *pfsr-x2-Ty1-glmS* and *pfsr-x2-Ty1-GFP* parasite lines, donor sequences comprising a 407-bp fragment from the 3′ coding region and a 457-bp fragment from the 3′ UTR of *pfsr-x2*, together with the Ty1-*glmS* or Ty1-GFP tag, were cloned into the pL6CS-WR vector between the *AflII* and *AscI* sites. Guide RNAs targeting *pfsr-x2* were inserted into the same vector between the *PstI* and *XhoI* sites. The resulting pL6CS-pfsr-x2-Ty1-*glmS* (or pL6CS-pfsr-x2-Ty1-GFP) donor plasmid and the Cas9 expression plasmid pUF1-Cas9 were introduced into fresh erythrocytes by electroporation (310 V, 950 µF). Transfected erythrocytes were immediately infected with Percoll-enriched schizont-stage parasites. Transfectants were maintained under dual drug selection (2 µg mL^−1^ blasticidin S (BSD) and 2.5 nM WR99210) until parasites were detectable by microscopic examination of Giemsa-stained blood smears. Genomic DNA was extracted, and correct integration was confirmed by diagnostic PCR followed by Sanger sequencing. After verification of the transgenic lines, clonal parasites of the *pfsr-x2-Ty1-glmS* line were obtained by limiting-dilution cloning.

### Parasite growth assay

Tightly synchronized ring-stage *pfsr-x2-Ty1-glmS* cultures were adjusted to 0.1% parasitemia, split into two groups in a six-well plate, and cultured in the presence or absence of 2.5 mM glucosamine (GlcN). Cultures were maintained continuously for four consecutive replication cycles. Parasitemia was quantified at each cycle by light microscopic examination of Giemsa-stained thin blood smears. All experiments were performed in triplicate, and data were analyzed using GraphPad Prism.

### Phylogenetic analysis

Protein sequences were obtained from the NCBI database. Multiple sequence alignment was performed using Clustal X2 with default parameters. A phylogenetic tree was constructed in MEGA12 using the neighbor-joining (NJ) method (Kumar et al., 2024). The resulting tree was visualized and edited using iTOL v5 (Interactive Tree of Life) (Letunic and Bork, 2021).

### Indirect immunofluorescence assay

Immunofluorescence analysis was performed essentially as described (Shang et al., 2021). Briefly, infected erythrocytes were treated with 0.15% saponin to lyse the host cells, and the released parasites were fixed with 4% paraformaldehyde on ice for 20 min. The fixed parasites were then allowed to settle onto microscope slides and processed for indirect immunofluorescence staining. Rabbit anti-GFP primary antibody (Abcam, ab290) was used at a dilution of 1:500, and Alexa Fluor 488-conjugated anti-rabbit secondary antibody (Thermo, A32732) was used at a dilution of 1:2000. After antibody staining, parasite nuclei were counterstained with DAPI (4′,6-diamidino-2-phenylindole) before imaging. Images were acquired using a 100× objective on an Olympus FV1000 confocal microscope. FV10-ASW software was used for image processing and figure preparation.

### Western blotting

Synchronized parasite cultures at 5% parasitemia were collected and lysed with 0.15% saponin. After lysis, parasites were washed with phosphate-buffered saline (PBS), resuspended in 1× SDS sample buffer, and separated on 10% SDS-polyacrylamide gels by electrophoresis. Proteins were then transferred onto membranes for western blot analysis. PfSR-X2, with an apparent molecular mass of approximately 36 kDa, was detected using a mouse anti-Ty1 antibody (Diagenode, C15200054). Rabbit anti-PfAldolase antibody (Abcam, ab207494) or anti-Histone H3 (Abcam, ab1791) were used as a loading control. Signals were detected using the ECL Prime Western Blotting Detection Reagent (GE Healthcare). The relative abundance of PfSR-X2 was normalized to PfAldolase levels using ImageJ software (NIH).

### RNA-seq and data analysis

Tightly synchronized *pfsr-x2-Ty1-glmS* parasite cultures were divided into two groups and incubated in the presence or absence of 2.5 mM GlcN. Total RNA was isolated at the ring (10 h post-invasion, hpi), trophozoite (30 hpi), and schizont (40 hpi) stages of the second intraerythrocytic developmental cycle. RNA extraction was performed using an RNA purification kit (Zymo Research, R1013) according to the manufacturer’s instructions. Strand-specific RNA-seq libraries were prepared with the VAHTS Universal V10 RNA-seq Library Prep Kit (NR606-01) and sequenced on an Illumina NovaSeq 6000 platform to generate 150 bp paired-end reads. Raw sequencing reads were trimmed using Trim Galore (v0.6.6) and aligned to the *Plasmodium falciparum* 3D7 reference genome (PlasmoDB-64_Pfalciparum3D7) with HISAT2 (v2.2.1). SAMtools (v1.12) was used for file conversion and duplicate read removal. The resulting sorted BAM files were converted to bigWig format using bamCoverage in deepTools (v3.5.4). Read counts were obtained using featureCounts (v2.0.1). Differentially expressed genes (DEGs) were identified using DESeq2 in R (v4.4.2) with thresholds of fold change > 1.5 and *P*< 0.05. Alternative splicing events were analyzed using rMATS (v4.0.2), and sashimi plots were generated using rmats2sashimiplots (v2.0.3).

### RNA immunoprecipitation and sequencing

RNA immunoprecipitation (RIP) assays were performed as described (Fan et al., 2020). Briefly, approximately 5 × 10^9^ tightly synchronized ring-stage or schizont-stage parasites were obtained by saponin lysis of infected erythrocytes. Parasite pellets were lysed under non-denaturing conditions in lysis buffer containing 50 mM Tris-HCl (pH 7.4), 150 mM NaCl, 1 mM EDTA, 1 mM EGTA, 1% Triton X-100/NP-40, 1× protease inhibitor cocktail, and 0.2 U μL^−1^ RNase inhibitor. After clarification, the supernatants were incubated with anti-GFP antibody or normal rabbit IgG, together with protein A/G magnetic beads. RNA associated with the immunocomplexes and input RNA samples were extracted using TRIzol reagent.

Input RNA was used directly for strand-specific RNA-seq library construction, whereas immunoprecipitated RNA was used for library preparation without poly(A) selection. Libraries were sequenced on an Illumina NovaSeq 6000 platform to generate 150 bp paired-end reads. RIP-seq raw reads were aligned to the reference genome using HISAT2 (v2.2.1). SAMtools (v1.12) was used for file conversion and removal of duplicate reads. The resulting sorted BAM files were converted to bigWig format with bamCoverage in deepTools (v3.5.4). Read counts were generated using featureCounts (v2.0.1). Differentially enriched genes were identified using DESeq2 in R (v4.4.2) with thresholds of |fold change| > 4 and *P* < 0.05. Peaks were called using MACS2 (v2.2.6) and annotated using ChIPseeker (v1.44.0) in R (v4.4.2). Enrichment profiles were generated using the computeMatrix and plotProfile functions in deepTools (v3.5.4).

### GO enrichment analysis

Gene Ontology (GO) enrichment analysis was performed for the selected genes using the GO enrichment tool available in PlasmoDB. GO terms with a *P* ≤ 0.05 were considered significantly enriched. The enrichment results were visualized in R using the ggplot2 package.

## Results

### Characterization of SR-X2 in P. falciparum

**Figure 1:**
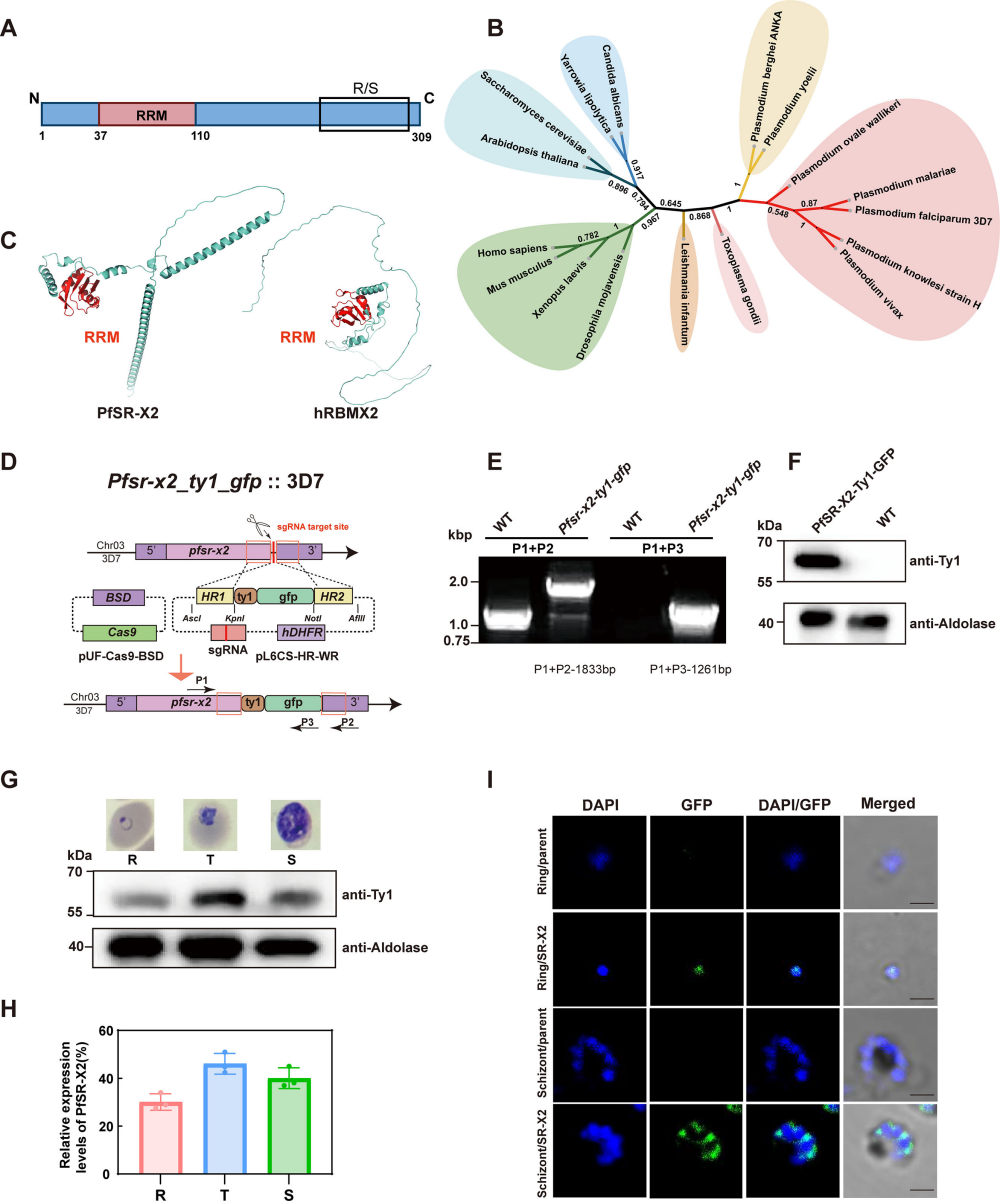
Characterization of the SR-X2 orthologue in *Plasmodium falciparum*. **(A)** Schematic representation of the domain architecture of PfSR-X2. The RNA-recognition motif (RRM) domain and the arginine/serine-rich region are indicated. **(B)** Phylogenetic analysis of SR-X2 orthologues from representative eukaryotic species. Bootstrap values are shown at the corresponding nodes, and different colors indicate major taxonomic groups. **(C)** AlphaFold-predicted tertiary structures of PfSR-X2 (AF-O97318-F1-model_v6) and human RBMX2 (AF-Q9Y388-F1-model_v6). The RRM domains are highlighted in red. **(D)** Schematic of the CRISPR/Cas9-mediated strategy used to generate the *pfsr-x2-ty1-gfp* transgenic parasite line. The positions of the sgRNA target site, homology regions, diagnostic PCR primers, and the Ty1-GFP tag are indicated. **(E)** Diagnostic PCR analysis confirming correct integration of the Ty1-GFP tag at the endogenous *pfsr-x2* locus. Primer pairs P1/P2 and P1/P3 were used to verify 5′ and 3′ integration, respectively. **(F)** Western blot analysis confirming expression of PfSR-X2-Ty1-GFP in the transgenic line. Aldolase was used as a loading control. **(G)** Stage-specific expression of PfSR-X2-Ty1-GFP in ring, trophozoite, and schizont stages. Representative parasite images are shown above the Western blot. Aldolase was used as a loading control. **(H)** Quantification of PfSR-X2-Ty1-GFP expression at different intraerythrocytic developmental stages, normalized to aldolase. Data are presented as mean ± SD from three independent experiments. **(I)** Indirect immunofluorescence assay showing the subcellular localization of PfSR-X2-Ty1-GFP in ring- and schizont-stage parasites. Parental 3D7 parasites were included as negative controls. Nuclei were stained with DAPI, and PfSR-X2-Ty1-GFP was detected by GFP fluorescence. Scale bars, 2 μm.

PfSR-X2 (PF3D7_0319500, PlasmoDB) comprises 309 amino acids, with residues 37–110 forming an RNA-recognition motif (RRM) domain (https://www.uniprot.org) and abundant serine and arginine residues are present within the C-terminal region (**Figure 1A**). To investigate a possible evolutionary conservation of PfSR-X2 among different eukaryotes, we compared the amino acid sequences of the RRM domains, which revealed that PfSR-X2 is more highly conserved among human-infecting *Plasmodium* species than in rodent malaria parasites or other eukaryotic organisms (**Figure 1B**; **Supplementary Figure S1A**; **Supplementary Table 1**). To assess the structural similarity between PfSR-X2 and its human counterpart hRBMX2, both proteins were modelled using AlphaFold. The predicted structures indicated that PfSR-X2 and hRBMX2 differ substantially at the structural level (**Figure 1C**).

To examine the expression pattern and subcellular localization of PfSR-X2 during the intraerythrocytic developmental cycle of *P. falciparum*, we generated a *pfsr-x2-Ty1-GFP* transgenic parasite line using the CRISPR-Cas9 genome editing system. The Ty1-GFP tag was introduced at the endogenous *pfsr-x2* locus through homologous recombination, allowing detection of PfSR-X2 under its native regulatory context (**Figure 1D**). Correct integration of the tagging cassette was verified by diagnostic PCR. The expected amplicons were detected using primer pairs P1 + P2 and P1 + P3 in the *pfsr-x2-Ty1-GFP* parasite line, with product sizes of 1833 bp and 1281 bp, respectively (**Figure 1E**). Western blot analysis using an anti-Ty1 antibody further confirmed successful tagging of PfSR-X2, as a specific band corresponding to PfSR-X2-Ty1-GFP was detected in the transgenic parasites but not in the wild-type control. Aldolase was used as a loading control (**Figure 1F**).

We next assessed the stage-specific expression of PfSR-X2 in tightly synchronized parasites collected at the ring, trophozoite, and schizont stages. Western blot analysis showed that PfSR-X2 was detectable at all three intraerythrocytic stages, indicating that the protein is expressed throughout asexual blood-stage development (**Figure 1G**). Densitometric quantification normalized to aldolase confirmed this expression pattern, with PfSR-X2 showing significantly higher expression at the trophozoite stage than at the ring and schizont stages (**Figure 1H**).

Finally, the subcellular distribution of PfSR-X2 was examined by indirect immunofluorescence assay. No GFP signal was observed in the parental control parasites, confirming the specificity of the fluorescence signal. In the *pfsr-x2-Ty1-GFP* parasites, PfSR-X2-associated GFP fluorescence was detected in both ring and schizont stages. In ring-stage parasites, the GFP signal appeared as a discrete focus that partially overlapped with, or was located adjacent to, the DAPI-stained nucleus. In schizont-stage parasites, PfSR-X2 showed a more abundant and dispersed distribution, with GFP signals observed both around multiple DAPI-stained nuclei and in extranuclear regions (**Figure 1I**). These observations indicate that PfSR-X2 localizes to both nuclear and cytoplasmic compartments during intraerythrocytic development, consistent with a potential role in RNA-associated regulatory processes.

### Engineering and phenotypic characterization of PfSR-X2 knockdown parasites

To investigate the functional importance of PfSR-X2 during the asexual blood stages of *P. falciparum*, we first attempted to disrupt the *pfsr-x2* open reading frame by CRISPR-Cas9-mediated insertion of a GFP-coding sequence. However, no viable parasites were recovered after three independent transfection attempts. Together with previous genome-wide mutagenesis data showing that *pfsr-x2* is refractory to disruption (Zhang et al., 2018), this result suggests that PfSR-X2 is likely required for asexual blood-stage growth, although alternative approaches could be used to further validate its indispensability.

**Figure 2:**
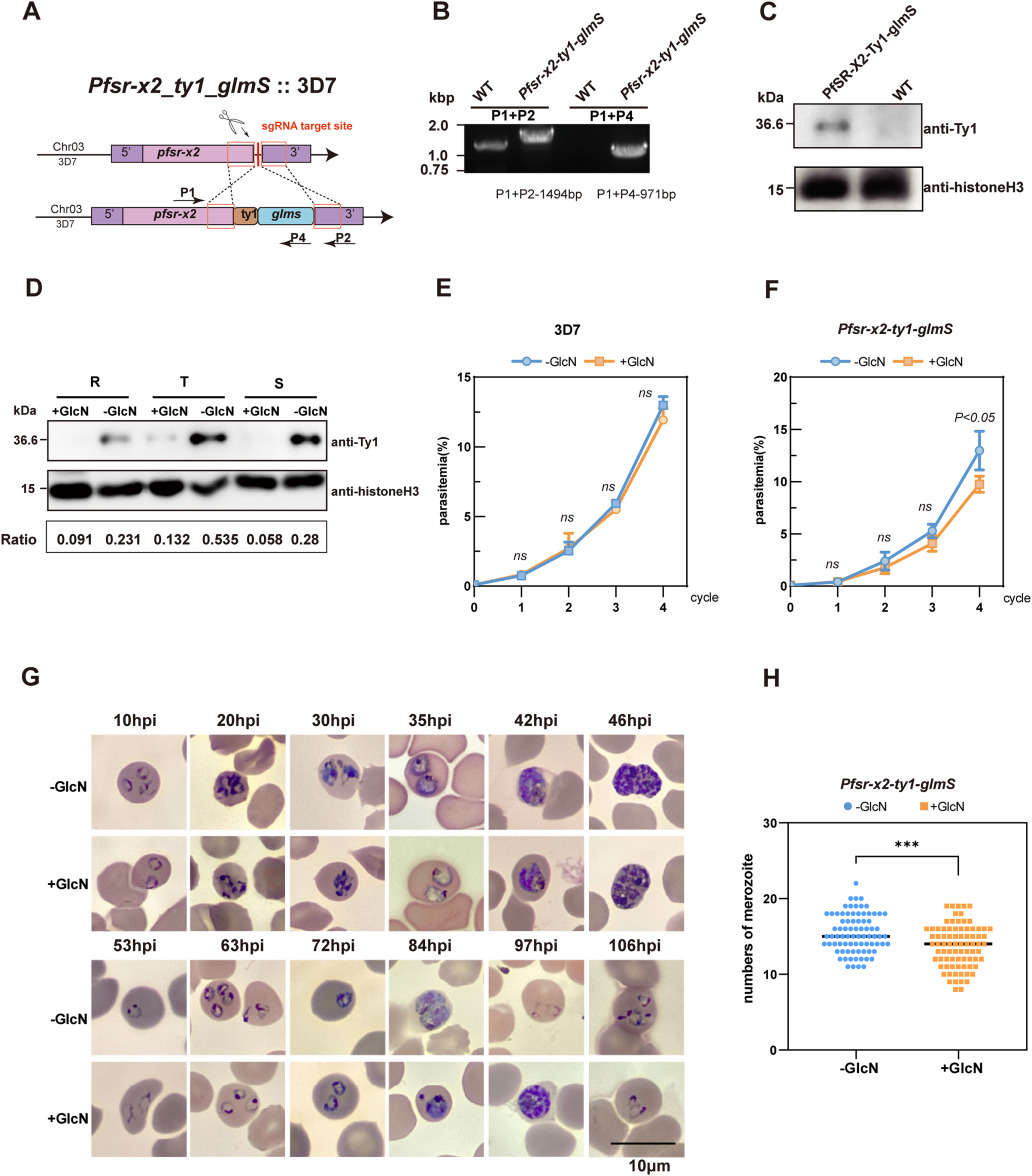
Generation and characterization of the *pfsr-x2*-Ty1-*glmS* knockdown strain. **(A)** Schematic representation of the strategy used to generate the *pfsr-x2*-Ty1-*glmS* transgenic parasite line. The sgRNA target site, homology regions, diagnostic PCR primers, Ty1 tag, and *glmS* ribozyme sequence are indicated. **(B)** Diagnostic PCR analysis confirming correct integration of the Ty1-*glmS* cassette at the endogenous *pfsr-x2* locus. Primer pairs P1/P2 and P1/P4 were used to verify the modified locus. **(C)** Western blot analysis confirming expression of PfSR-X2-Ty1-*glmS* in the transgenic line. PfSR-X2-Ty1-*glmS* was detected using an anti-Ty1 antibody, and histone H3 was used as a loading control. **(D)** Western blot analysis of PfSR-X2-Ty1-*glmS* protein levels in ring, trophozoite, and schizont-stage parasites cultured in the absence or presence of glucosamine. PfSR-X2-Ty1-*glmS* was detected using an anti-Ty1 antibody, and histone H3 was used as a loading control. The values below the blots indicate the relative band intensities of PfSR-X2-Ty1-*glmS* normalized to histone H3. **(E, F)** Asexual blood-stage growth curves of the parental 3D7 line **(E)** and the *pfsr-x2*-Ty1-*glmS* knockdown line **(F)** cultured in the absence or presence of glucosamine for four replication cycles. Parasitemia is presented as mean ± SD. ns, not significant; *P* < 0.05. **(G)** Giemsa-stained thin blood smears showing representative parasite morphology of the *pfsr-x2*-Ty1-*glmS* line cultured in the absence or presence of glucosamine at the indicated time points post-invasion. Scale bar, 10 μm. **(H)** Quantification of merozoite numbers per schizont in the *pfsr-x2*-Ty1-*glmS* line cultured in the absence or presence of glucosamine. Each dot represents an individual schizont; bars indicate mean ± SD; *n* = 80 schizonts per group. ***P < 0.001. Statistical significance in (E, F, H) was determined using Student’s *t*-test.

Because complete disruption of *pfsr-x2* was not tolerated, we generated a conditional knockdown parasite line by integrating a Ty1 epitope tag followed by a glucosamine-inducible *glmS* ribozyme into the endogenous *pfsr-x2* locus (Prommana et al., 2013). In this system, addition of glucosamine induces ribozyme-mediated cleavage of the target mRNA, allowing post-transcriptional depletion of PfSR-X2. The *pfsr-x2-Ty1-glmS* parasite line was generated by CRISPR-Cas9-mediated homologous recombination using the pL6CS-pfsr-x2-Ty1-*glmS* donor plasmid together with the pUF-Cas9 plasmid. The donor cassette was designed to introduce the Ty1-*glmS* sequence at the 3′ end of *pfsr-x2*, thereby preserving expression from the endogenous promoter while enabling conditional knockdown upon GlcN treatment (**Figure 2A**). Drug selection with blasticidin S (BSD) and WR99210 was applied after transfection, and viable transgenic parasites were obtained after approximately 25 days.

Correct integration of the Ty1-*glmS* cassette was first verified by diagnostic PCR using primer pairs spanning the modified locus. The expected PCR products were detected in the *pfsr-x2-Ty1-glmS* parasite line, confirming successful modification of the endogenous *pfsr-x2* locus (**Figure 2B**). Western blot analysis using an anti-Ty1 antibody further confirmed expression of the tagged PfSR-X2 protein in the transgenic parasites, while no Ty1 signal was detected in the wild-type 3D7 line. Histone H3 was used as a loading control (**Figure 2C**). Following confirmation of correct integration, clonal parasite lines were obtained by limiting dilution.

To evaluate the efficiency of conditional knockdown, synchronized *pfsr-x2-Ty1-glmS* parasites were treated with 2.5 mM GlcN and collected at representative ring, trophozoite, and schizont stages. Western blotting showed that GlcN treatment markedly reduced PfSR-X2 protein abundance at all three stages compared with untreated controls (**Figure 2D**). Quantification of the Ty1 signal normalized to histone H3 showed that PfSR-X2 levels were reduced from 0.231 to 0.091 at the ring stage, from 0.535 to 0.132 at the trophozoite stage, and from 0.280 to 0.058 at the schizont stage after GlcN treatment.

We next examined whether PfSR-X2 depletion affects parasite proliferation. In the parental 3D7 line, addition of GlcN did not significantly alter parasite growth over four consecutive intraerythrocytic cycles (**Figure 2E**). In contrast, the *pfsr-x2-Ty1-glmS* line showed a progressive growth defect upon GlcN treatment. Although no significant difference was observed during the first three cycles, parasitemia was significantly reduced in GlcN-treated knockdown parasites by the fourth cycle compared with untreated controls (**Figure 2F**). These data indicate that partial depletion of PfSR-X2 compromises parasite proliferation, particularly after prolonged culture.

To further characterize the developmental consequences of PfSR-X2 depletion, Giemsa-stained blood smears were examined across the intraerythrocytic developmental cycle. Both untreated and GlcN-treated *pfsr-x2-Ty1-glmS* parasites were able to progress from ring to trophozoite and schizont stages, suggesting that PfSR-X2 depletion did not cause an immediate or complete developmental arrest (**Figure 2G**; **Supplementary Figure S1B**). However, GlcN-treated parasites displayed impaired schizont maturation, as reflected by a reduced number of merozoites per mature schizont. Quantitative analysis confirmed that PfSR-X2 knockdown significantly decreased the number of merozoites produced per schizont compared with untreated parasites (**Figure 2H**). Together, these results suggest that PfSR-X2 is important for efficient asexual replication and may contribute to optimal schizont maturation or daughter merozoite formation.

### PfSR-X2 depletion reshapes the stage-specific transcriptome of P. falciparum

**Figure 3:**
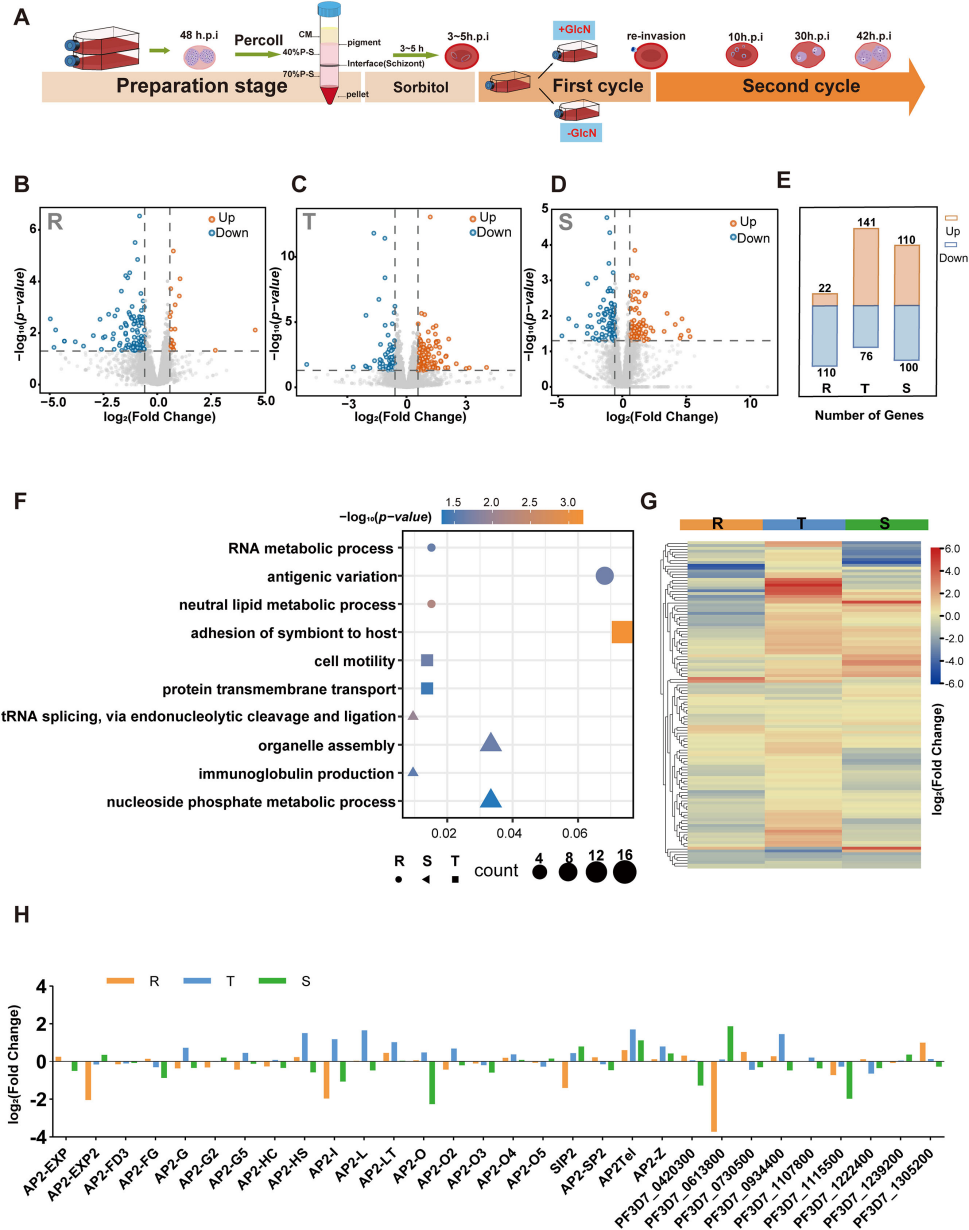
PfSR-X2 knockdown alters the transcriptome of asexual blood-stage parasites. **(A)** Schematic overview of the RNA-seq sample collection strategy. Highly synchronized *pfsr-x2*-Ty1-*glmS* parasites were cultured in the absence or presence of glucosamine during the first replication cycle, and total RNA was collected from ring, trophozoite, and schizont-stage parasites during the second intraerythrocytic developmental cycle. Three biological replicates were prepared for each condition. **(B–D)** Volcano plots showing differentially expressed genes in PfSR-X2 knockdown parasites cultured with glucosamine compared with those cultured without glucosamine at the ring **(B)**, trophozoite **(C)**, and schizont **(D)** stages. Orange and blue dots indicate significantly upregulated and downregulated genes, respectively; grey dots indicate genes that were not significantly changed. Differentially expressed genes were defined using thresholds of *P* value < 0.05 and |fold change| > 1.5. Dashed lines indicate the thresholds used for differential-expression analysis. **(E)** Summary of the numbers of upregulated and downregulated genes identified at the ring, trophozoite, and schizont stages. Orange and blue bars represent upregulated and downregulated genes, respectively. **(F)** Gene Ontology biological process enrichment analysis of differentially expressed genes following PfSR-X2 knockdown across the intraerythrocytic developmental cycle. Dot size indicates the number of genes enriched in each term, and color intensity represents the significance of enrichment, shown as -log_10_(*P* value). Different symbols indicate the parasite stage associated with each enriched term. **(G)** Heatmap showing the expression changes of *rif* genes in PfSR-X2 knockdown parasites cultured with glucosamine compared with those cultured without glucosamine at the ring, trophozoite, and schizont stages. The color scale represents log_2_(fold change). **(H)** Expression changes of ApiAP2 transcription factor family genes in PfSR-X2 knockdown parasites cultured with glucosamine compared with those cultured without glucosamine at different intraerythrocytic developmental stages. Bars represent log_2_(fold change) values at the ring, trophozoite, and schizont stages.

To investigate whether PfSR-X2 contributes to transcriptional regulation during the asexual blood-stage development of *P. falciparum*, comparative RNA-seq analysis was performed using tightly synchronized *pfsr-x2-Ty1-glmS* parasites cultured in the presence or absence of GlcN. Highly synchronized parasites were first enriched by Percoll purification and sorbitol treatment, and GlcN was added at the beginning of the first intraerythrocytic developmental cycle. Parasites were then allowed to reinvade, and samples were collected during the second cycle at representative ring, trophozoite, and schizont stages, corresponding to 10, 30, and 42 h post-invasion, respectively (**Figure 3A**). RNA-seq analysis revealed that PfSR-X2 depletion caused marked but stage-dependent changes in parasite gene expression. Volcano plot analysis showed distinct sets of differentially expressed genes (DEGs) at the ring, trophozoite, and schizont stages, with both upregulated and downregulated transcripts detected after GlcN-induced PfSR-X2 knockdown (**Figures 3B–D**; **Supplementary Table 2**). At the ring stage, 22 genes were upregulated and 110 genes were downregulated. At the trophozoite stage, the number of upregulated genes increased substantially, with 141 upregulated and 76 downregulated genes detected. At the schizont stage, 110 genes were upregulated and 100 genes were downregulated, suggesting a more balanced transcriptional response during late intraerythrocytic development (**Figure 3E**; **Supplementary Table 2**).

To gain insight into the biological processes affected by PfSR-X2 depletion, Gene Ontology enrichment analysis was performed for the DEGs identified at each developmental stage. At the ring stage, enriched terms included RNA metabolic process, antigenic variation, and neutral lipid metabolic process. At the trophozoite stage, enriched terms were mainly associated with adhesion of symbiont to host, cell motility, and protein transmembrane transport. At the schizont stage, enriched terms included tRNA splicing via endonucleolytic cleavage and ligation, organelle assembly, immunoglobulin production, and nucleoside phosphate metabolic process, indicating that PfSR-X2 depletion may influence RNA processing and late-stage cellular organization (**Figure 3F**). These enrichment patterns further support a stage-dependent role for PfSR-X2 in maintaining parasite gene expression programs.

Among the DEGs, members of antigenically variant gene families were prominently affected. In particular, the *rif* multigene family displayed extensive transcriptional changes across the ring, trophozoite, and schizont stages, as shown by hierarchical clustering of log_2_ fold-change values (**Figure 3G**). The heatmap revealed that many *rif* genes were differentially regulated in a stage-dependent manner, with the trophozoite stage showing a particularly strong transcriptional response. In contrast, the overall expression pattern of *var* genes remained largely unchanged among the three single clones examined (**Supplementary Figure 2**). These results suggest that PfSR-X2 depletion preferentially affects a subset of antigenic variation-associated genes, especially the *rif* family, rather than causing a broad and uniform disruption of all variant surface antigen gene families.

Given the extensive transcriptomic changes observed after PfSR-X2 depletion, we next examined whether genes encoding known transcriptional regulators were affected. The ApiAP2 family plays a central role in controlling stage-specific transcriptional programs in *P. falciparum* (Shang et al., 2022; Singhal et al., 2024). Analysis of RNA-seq data showed that multiple ApiAP2 transcription factor genes exhibited altered expression upon PfSR-X2 knockdown, and these changes varied across the ring, trophozoite, and schizont stages (**Figure 3H**). Several ApiAP2 genes showed stage-restricted upregulation or downregulation, suggesting that PfSR-X2 depletion may indirectly or directly influence transcriptional regulatory networks.

Because PfSR-X2 contains an RNA-recognition motif and shows similarity to RNA-binding proteins, we further assessed whether its depletion affects pre-mRNA splicing. Alternative splicing analysis of the RNA-seq data using rMATS identified only limited splicing alterations at the ring and trophozoite stages. At the ring stage, three candidate splicing events were detected, but none remained significant after FDR correction. At the trophozoite stage, nine candidate events, including skipped exon, mutually exclusive exon, alternative 5′ splice site, and retained intron events, were identified, but these also did not pass the FDR threshold. In contrast, the schizont stage exhibited the most pronounced splicing response, with 18 candidate differential splicing events detected, of which nine remained significant after multiple-testing correction. These significant events mainly consisted of skipped exon and mutually exclusive exon events, together with two alternative 3′ splice site events (**Supplementary Table 3**). These results suggest that PfSR-X2 depletion has a limited but stage-dependent effect on alternative splicing, with the strongest effect observed during schizont development.

### Identification of PfSR-X2-associated transcripts by RIP-seq

**Figure 4:**
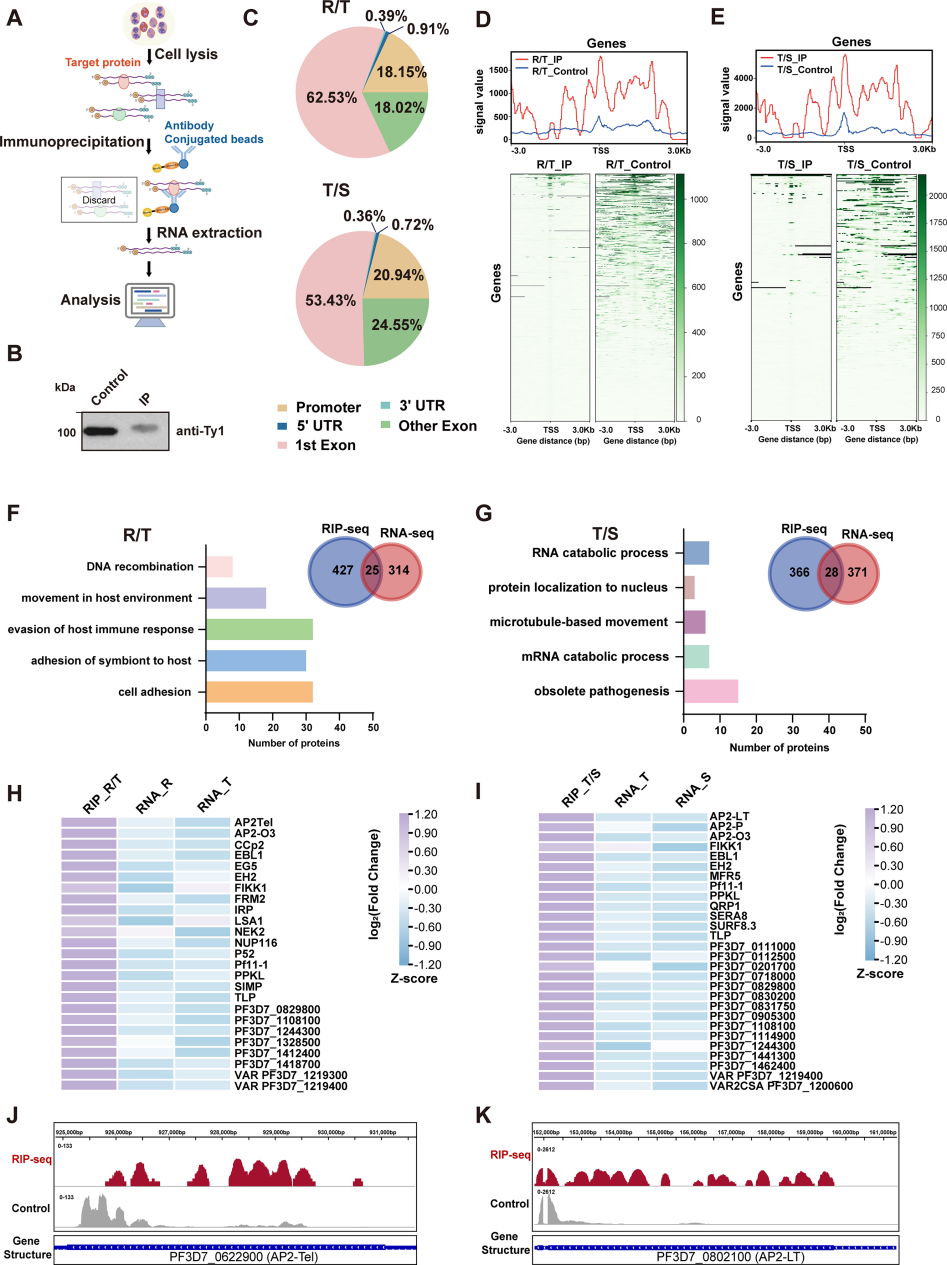
Genome-wide analysis of PfSR-X2-associated target RNAs. **(A)** Schematic representation of the RNA immunoprecipitation sequencing (RIP-seq) workflow used to identify PfSR-X2-associated RNAs. PfSR-X2-Ty1-GFP-associated ribonucleoprotein complexes were immunoprecipitated using antibody-conjugated beads, followed by RNA extraction and sequencing analysis. **(B)** Western blot analysis validating the immunoprecipitation efficiency of PfSR-X2-Ty1-GFP. PfSR-X2-Ty1-GFP was detected using an anti-Ty1 antibody. Control, input/control sample; IP, immunoprecipitated sample. **(C)** Genomic distribution of PfSR-X2 RIP-seq peaks at the ring-to-trophozoite stage transition, R/T, 15–30 h post-invasion, and the trophozoite-to-schizont stage transition, T/S, 30–42 h post-invasion. Peaks were assigned to promoter regions, 5′ UTRs, first exons, 3′ UTRs, and other exonic regions. **(D, E)** Metagene profiles and heatmaps showing PfSR-X2 RIP-seq signal enrichment around transcription start sites at the R/T **(D)** and T/S **(E)** stages. Signal intensity is shown within ±3 kb of the transcription start site. Red lines indicate RIP-seq immunoprecipitation samples, and blue lines indicate the corresponding control samples. **(F, G)** Gene Ontology biological process enrichment analysis of genes encoding PfSR-X2-associated transcripts at the R/T **(F)** and T/S **(G)** stages. Bar plots show the number of genes assigned to each enriched term. Venn diagrams indicate the overlap between genes identified by RIP-seq and differentially expressed genes identified by RNA-seq after PfSR-X2 knockdown. **(H, I)** Heatmaps showing PfSR-X2 RIP-seq enrichment and corresponding RNA-seq expression changes for overlapping genes identified in both RIP-seq and RNA-seq datasets at the R/T **(H)** and T/S **(I)** stages. Color intensity represents Z-score-transformed log_2_(fold change) values. **(J, K)** Representative genome-browser tracks showing PfSR-X2 RIP-seq enrichment at selected ApiAP2 transcription factor loci, including PF3D7_0622900/PfAP2-Tel **(J)** and PF3D7_0802100/PfAP2-LT **(K)**. RIP-seq signals are shown in red, control signals are shown in grey, and gene structures are shown below each track.

To identify RNA transcripts associated with PfSR-X2 in *P. falciparum*, RNA immunoprecipitation followed by sequencing was performed using the *pfsr-x2-Ty1-GFP* parasite line. Tightly synchronized parasites were collected at two developmental windows, corresponding to the ring-to-trophozoite stage (R/T) and trophozoite-to-schizont stage (T/S). Native parasite lysates were subjected to immunoprecipitation (IP) using antibody-conjugated beads, and the co-IP RNA was extracted for strand-specific RIP-seq library construction (**Figure 4A**). Western blot analysis using an anti-Ty1 antibody confirmed the recovery of Ty1-tagged PfSR-X2 in the immunoprecipitated fraction, supporting the specificity of the RIP assay (**Figure 4B**).

Peak annotation showed that PfSR-X2-associated RNA fragments were strongly enriched within exonic regions. In the R/T dataset, 62.53% of RIP-seq peaks were located within exons. A similar distribution was observed in the T/S dataset, in which 53.43% of peaks were located within exons (**Figure 4C**). These results indicate that PfSR-X2 preferentially associates with exon-containing transcript regions. Metagene analysis further showed that RIP-seq signals were markedly enriched in the PfSR-X2 immunoprecipitated samples compared with the corresponding controls. In both the R/T and T/S datasets, PfSR-X2-associated signals were distributed across transcript regions downstream of the transcription start site, with substantially stronger enrichment in the RIP samples than in the control samples (**Figures 4D, E**). Heatmap visualization of RIP-seq signals across target genes further confirmed the reproducible enrichment of PfSR-X2-associated RNA fragments in the immunoprecipitated samples.

We next performed motif enrichment analysis to explore whether PfSR-X2-associated RNA fragments contained preferred sequence features. Distinct enriched motifs were identified in the two developmental windows. In the R/T dataset, the top-ranked enriched motifs included KGARCAASACTA, AGGGAACG, and AGGTGACCTC. In the T/S dataset, the predominant motifs included GAGACATAGC, GTTATTGGGGAB, and TCATCTAGTCCA (**Supplementary Figure 3A**). Functional enrichment analysis was then performed on the PfSR-X2-associated transcripts. In the R/T dataset, PfSR-X2-associated genes were enriched in biological processes related to cell adhesion, adhesion of symbiont to host, evasion of host immune response, movement in host environment, and DNA recombination (**Figure 4F**). In the T/S dataset, enriched terms included RNA catabolic process, protein localization to nucleus, microtubule-based movement, mRNA catabolic process, and pathogenesis-related processes (**Figure 4G**).

To determine whether PfSR-X2-associated transcripts were also affected by PfSR-X2 depletion, we integrated the RIP-seq data with the RNA-seq dataset generated from the *pfsr-x2-Ty1-glmS* knockdown parasites. In the R/T dataset, RIP-seq identified 452 PfSR-X2-associated transcripts, among which 25 overlapped with DEGs detected by RNA-seq. In the T/S dataset, 394 PfSR-X2-associated transcripts were identified, 28 of which overlapped with DEGs (**Figures 4F, G**; **Supplementary Table 4**). Heatmap analysis of the overlapping RIP-seq and RNA-seq targets showed that these transcripts were enriched in the RIP-seq datasets and displayed stage-dependent changes in transcript abundance following PfSR-X2 knockdown (**Figures 4H, I**). Notably, the overlapping targets included genes encoding antigenically variant proteins, exported proteins, and several ApiAP2 transcription factors, including *pfap2-tel*, *pfap2-o3*, *pfap2-p*, and *pfap2-lt*. Genome browser visualization further confirmed prominent PfSR-X2 RIP-seq enrichment over representative ApiAP2 genes, including *pfap2-tel* and *pfap2-lt*, whereas the corresponding control tracks showed much weaker signals (**Figures 4J, K**). Additional representative targets, including *pfemma1* and *pfvar2csa*, also showed clear RIP-seq enrichment compared with the control samples (**Supplementary Figures 3B, C**).

To further validate the RIP-seq results, RIP-qPCR was performed for selected candidate targets. Significant enrichment was detected for *pfap2-tel*, *pfap2-lt*, *pfemma1*, and *pfvar2csa* in the immunoprecipitated samples compared with the control samples (**Supplementary Figures 3D–G**). These validation results support the reliability of the RIP-seq dataset and confirm that PfSR-X2 associates with selected transcripts involved in transcriptional regulation and antigenic variation-associated processes.

Collectively, these results demonstrate that PfSR-X2 associates with a defined subset of parasite transcripts in a stage-dependent manner. The integration of RIP-seq and RNA-seq data suggests that PfSR-X2 may contribute to post-transcriptional regulation of genes involved in antigenic variation, host-parasite interactions, RNA metabolism, and ApiAP2-mediated transcriptional regulatory networks during the intraerythrocytic development of *P. falciparum*.

## Discussion

RNA-binding proteins are central components of post-transcriptional gene regulation in eukaryotes, but the functional diversity of SR-related proteins in *Plasmodium falciparum* remains incompletely understood. In this study, we characterized PfSR-X2, an RRM-containing protein with SR-like features, during the intraerythrocytic developmental cycle of *P. falciparum*. PfSR-X2 is conserved among human-infecting *Plasmodium* species, expressed throughout asexual blood-stage development, and distributed in both nuclear and extranuclear compartments. The functional significance of this dual localization remains to be explored, and future studies using compartment-specific approaches will be required to dissect the roles of nuclear versus cytoplasmic PfSR-X2. Conditional depletion of PfSR-X2 resulted in reduced parasite proliferation and impaired merozoite production. These findings indicate that PfSR-X2 is required for efficient asexual replication and suggest that it supports developmental processes that are important for sustained blood-stage growth.

A major finding of this study is that PfSR-X2 depletion caused broad, stage-dependent changes in transcript abundance. The affected genes were not randomly distributed, but were enriched in biological processes related to RNA metabolism, antigenic variation, host-parasite interactions, protein transport, and late-stage cellular organization. Among these changes, the transcriptional alteration of the *rif* multigene family was particularly prominent, whereas the overall *var* transcriptional pattern remained largely unchanged. This suggests that PfSR-X2 does not globally disrupt antigenic variation programs, but instead preferentially affects a subset of variant antigen-associated genes. In addition, multiple ApiAP2 transcription factor genes were differentially expressed following PfSR-X2 knockdown. Because ApiAP2 proteins are key regulators of stage-specific transcriptional programs in *P. falciparum*, changes in their transcript abundance may contribute to secondary transcriptional effects observed after PfSR-X2 depletion. Thus, PfSR-X2 may influence parasite gene-expression programs both through association with specific transcripts and through indirect effects on transcriptional regulatory networks.

Although SR proteins are classically known as regulators of pre-mRNA splicing, our data do not support a role for PfSR-X2 as a global alternative splicing factor during asexual blood-stage development. Only a small number of candidate splicing events were detected after PfSR-X2 knockdown, and significant changes after multiple-testing correction were mainly observed at the schizont stage. Therefore, the splicing-related effect of PfSR-X2 appears to be limited and stage-dependent rather than widespread. This distinction is important because several previously studied *Plasmodium* SR or SR-related proteins have been linked more directly to alternative splicing regulation. For example, PfSR1 overexpression alters alternative splicing activity and reduces asexual parasite growth (Goyal et al., 2021), whereas PbSR-MG is required for sex-specific splicing events during gametocyte development and transmission (Yeoh et al., 2019). In contrast, the primary consequence of PfSR-X2 depletion in our study was a marked disturbance of transcript abundance, especially among *rif* genes, ApiAP2 transcription factor genes, and host-parasite interaction-associated transcripts. These differences suggest that PfSR-X2 is functionally distinct from previously characterized *Plasmodium* SR proteins and may act mainly as a selective RNA-associated regulator rather than as a broad splice-regulatory factor.

This interpretation is further supported by the RIP-seq analysis. PfSR-X2 was associated with a defined subset of parasite transcripts in a stage-dependent manner, with enrichment mainly over exon-containing regions. Integration of RIP-seq and RNA-seq data identified candidate PfSR-X2-associated transcripts whose abundance was altered after PfSR-X2 depletion. These included transcripts encoding antigenically variant proteins, exported proteins, and ApiAP2 transcription factors such as PfAP2-Tel, PfAP2-O3, PfAP2-P, and PfAP2-LT. RIP-qPCR validation confirmed enrichment of selected targets, including PfAP2-Tel, PfAP2-LT, PfEMMA1, and PfVAR2CSA, supporting the reliability of the RIP-seq dataset. These results provide a mechanistic link between PfSR-X2 RNA association and the transcriptomic changes observed after knockdown, although further studies will be needed to determine whether PfSR-X2 directly stabilizes these transcripts, affects their processing, regulates their nuclear export, or participates in RNA-protein complexes that influence their fate.

The present study therefore extends current knowledge of *Plasmodium* SR-related proteins in several ways. First, PfSR-X2 was examined under endogenous expression conditions using epitope-tagged and conditional knockdown parasite lines, allowing its function to be assessed during the asexual blood-stage cycle. Second, the results reveal that PfSR-X2 is essential or near-essential for optimal parasite replication and merozoite formation. Third, rather than identifying PfSR-X2 as another general alternative splicing factor, our data suggest that it contributes mainly to stage-specific post-transcriptional regulation of selected transcripts, including those linked to antigenic variation and ApiAP2-mediated developmental control. Finally, the combination of conditional knockdown transcriptomics and RIP-seq provides a set of candidate PfSR-X2-associated regulatory targets, offering a resource for future studies on RNA-based regulation in malaria parasites.

There are several limitations to this study. The alternative splicing changes detected after PfSR-X2 depletion were relatively modest, and the functional importance of individual splicing events remains to be experimentally validated. Moreover, although PfSR-X2 is predicted to be homologous to core retention and splicing (RES) complex components (Dziembowski et al., 2004; Fernandez et al., 2018), physical interaction with other putative RES homologs in *P. falciparum* was not experimentally verified in this study. The observed intron retention and splicing defects upon PfSR-X2 depletion are consistent with a compromised RES function, yet future biochemical approaches such as co-immunoprecipitation will be needed to confirm its integration into a RES-like complex. In addition, RIP-seq identifies transcripts associated with PfSR-X2-containing ribonucleoprotein complexes, but it does not define the precise binding sites or distinguish direct RNA binding from indirect association through other proteins. Future studies using higher-resolution approaches, such as CLIP-seq, together with transcript stability assays and target-specific functional validation, will be required to clarify how PfSR-X2 regulates its associated transcripts. Although only a subset of PfSR-X2-bound transcripts overlap with differentially expressed genes, PfSR-X2 may regulate other targets post-transcriptionally, such as through effects on RNA stability, processing, or translation. Future studies will be needed to determine the functional consequences for these bound but non-differentially expressed transcripts.

In summary, our findings identify PfSR-X2 as an essential RNA-associated regulator required for normal asexual blood-stage development of *P. falciparum*. Unlike a canonical global splicing factor, PfSR-X2 appears to exert a selective and stage-dependent influence on RNA metabolism, with prominent effects on transcripts involved in antigenic variation, host-parasite interactions, and ApiAP2-associated transcriptional regulation. These results highlight a previously uncharacterized layer of post-transcriptional control in malaria parasites and provide new insight into how RNA-binding proteins contribute to parasite development and antigenic variation-associated gene expression.

## Data Availability

The datasets presented in this study can be found in online repositories. The names of the repository/repositories and accession number(s) can be found in the article/**Supplementary Material**.

## Supplementary material

Supplementary Figure 1 Sequence alignment of PfSR-X2 and morphology of parental 3D7 parasites cultured with glucosamine. **(A)** Multiple sequence alignment of PfSR-X2 from *Plasmodium falciparum* with homologous proteins from *Plasmodium yoelii*, *Homo sapiens*, and *Saccharomyces cerevisiae*. The RNA-recognition motif (RRM) domain is indicated by the red box. **(B)** Representative Giemsa-stained thin blood smears showing the morphology of parental 3D7 parasites cultured in the absence or presence of glucosamine at the indicated time points post-invasion. Scale bar, 10 μm.

Supplementary Figure 2 Expression profiles of *var* genes in independent *pfsr-x2-Ty1-glmS* clones. **(A–C)** RNA-seq analysis of *var* gene expression in three independent *pfsr-x2-Ty1-glmS* clones, D10 **(A)**, F6 **(B)**, and B4 **(C)**, cultured in the absence or presence of glucosamine.

Supplementary Figure 3 Stage-specific enriched motifs and validation of PfSR-X2-associated RNAs. **(A)** Top enriched sequence motifs identified from PfSR-X2 RIP-seq peaks at the ring-to-trophozoite (R/T) and trophozoite-to-schizont (T/S) stages. The top three motifs at each stage are ranked according to statistical significance, and the corresponding -log_10_(P) values are indicated. **(B, C)** Representative genome browser views showing PfSR-X2 RIP-seq enrichment at selected loci, including PF3D7_1134300 (PfEMMA1) **(B)** and PF3D7_1200600 (PfVAR2CSA) **(C)**. RIP-seq signals are shown in red, control signals in grey, and gene structures are shown below each track. **(D–G)** RIP-qPCR validation of selected PfSR-X2-associated transcripts. Enrichment levels of *pfap2-tel* **(D)**, *pfap2-lt* **(E)**, *pfemma1* **(F)**, and *pfvar2csa* **(G)** transcripts in immunoprecipitated (IP) samples relative to control samples are shown. Data are presented as mean ± SD. *P* < 0.01.

Supplementary Table 1 Protein IDs of other SR-X2 proteins in eukaryotes and the sequences of the PCR primers used in the experiments.

Supplementary Table 2 DEGs of the *pfsr-x2-Ty1-glm*S strain with or without GlcN.

Supplementary Table 3 Analysis of alternative splicing in the *pfsr-x2-Ty1-glmS* strain with or without GlcN.

Supplementary Table 4 Target genes bound by PfSR-X2.
