# Supplementary figures and images for: The serine-arginine-rich protein PfSR-X2 modulates human malaria parasite gene expression during the intraerythrocytic developmental cycle
Source: Front Cell Infect Microbiol. 2026 May 26;16:1842355. doi: 10.3389/fcimb.2026.1842355 (PMC13247540; doi:10.3389/fcimb.2026.1842355)

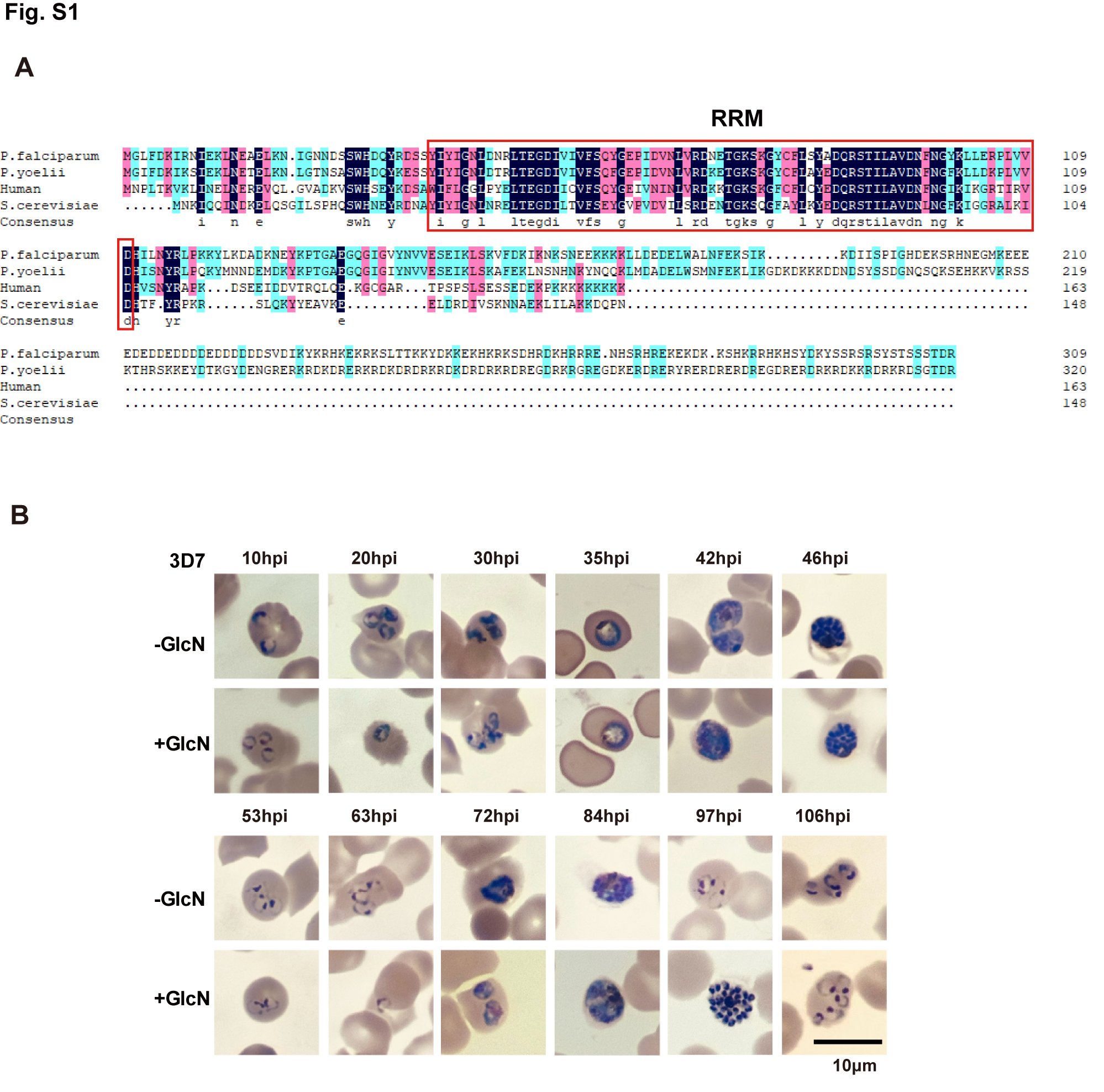

Supplement: Supplementary Figure 1 — Sequence alignment of PfSR-X2 and morphology of parental 3D7 parasites cultured with glucosamine. (A) Multiple sequence alignment of PfSR-X2 from Plasmodium falciparum with homologous proteins from Plasmodium yoelii, Homo sapiens, and Saccharomyces cerevisiae. The RNA-recognition motif (RRM) domain is indicated by the red box. (B) Representative Giemsa-stained thin blood smears showing the morphology of parental 3D7 parasites cultured in the absence or presence of glucosamine at the indicated time points post-invasion. Scale bar, 10 μm. [file Image1.tif]

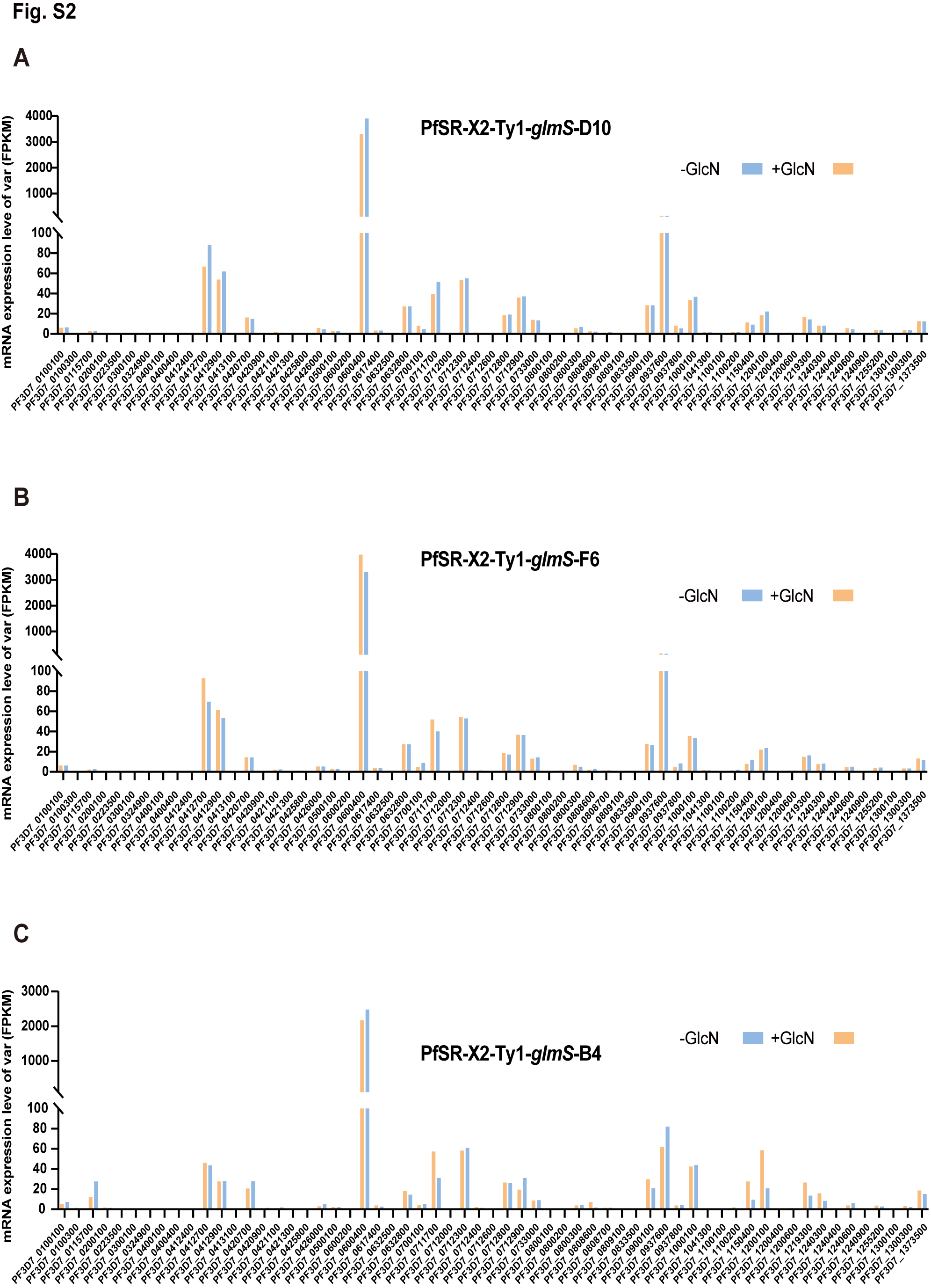

Supplement: Supplementary Figure 2 — Expression profiles of var genes in independent pfsr-x2-Ty1-glmS clones. (A–C) RNA-seq analysis of var gene expression in three independent pfsr-x2-Ty1-glmS clones, D10 (A), F6 (B), and B4 (C), cultured in the absence or presence of glucosamine. [file Image2.tif]

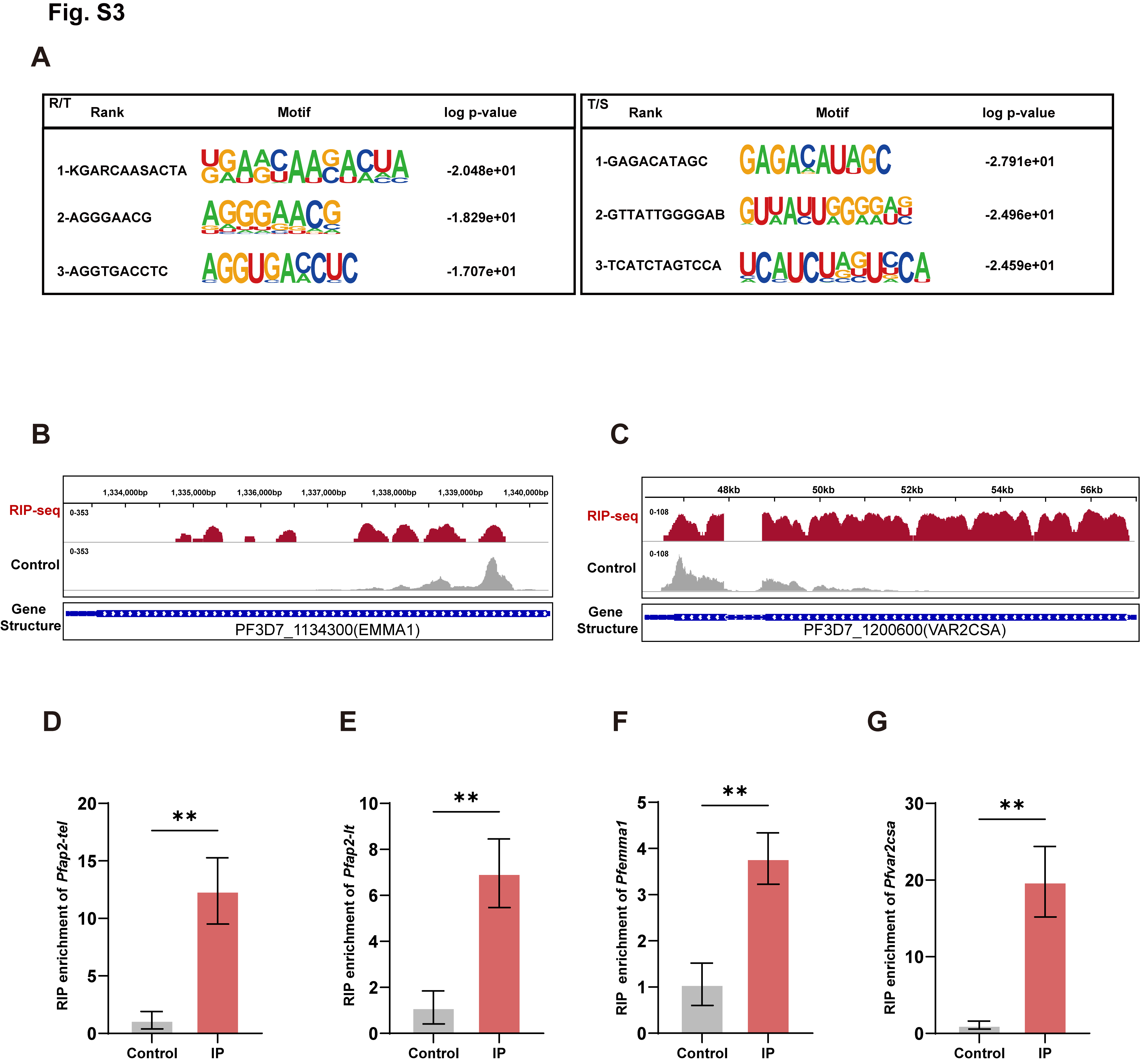

Supplement: Supplementary Figure 3 — Stage-specific enriched motifs and validation of PfSR-X2-associated RNAs. (A) Top enriched sequence motifs identified from PfSR-X2 RIP-seq peaks at the ring-to-trophozoite (R/T) and trophozoite-to-schizont (T/S) stages. The top three motifs at each stage are ranked according to statistical significance, and the corresponding -log10(P) values are indicated. (B, C) Representative genome browser views showing PfSR-X2 RIP-seq enrichment at selected loci, including PF3D7_1134300 (PfEMMA1) (B) and PF3D7_1200600 (PfVAR2CSA) (C). RIP-seq signals are shown in red, control signals in grey, and gene structures are shown below each track. (D–G) RIP-qPCR validation of selected PfSR-X2-associated transcripts. Enrichment levels of pfap2-tel (D), pfap2-lt (E), pfemma1 (F), and pfvar2csa (G) transcripts in immunoprecipitated (IP) samples relative to control samples are shown. Data are presented as mean ± SD. P < 0.01. [file Image3.tif]
